# SNPmplexViewer--toward a cost-effective traceability system

**DOI:** 10.1186/1756-0500-4-146

**Published:** 2011-05-23

**Authors:** Yanir Seroussi, Andrey Shirak, Eyal Seroussi

**Affiliations:** 1 Clayton School of Information Technology, Monash University, Clayton, Victoria 3800, Australia; 2 The Agricultural Research Organization, Institute of Animal Science, P.O. Box 6, Bet-Dagan 50250, Israel

## Abstract

**Background:**

Beef traceability has become mandatory in many regions of the world and is typically achieved through the use of unique numerical codes on ear tags and animal passports. DNA-based traceability uses the animal's own DNA code to identify it and the products derived from it. Using *SNaPshot*, a primer-extension-based method, a multiplex of 25 SNPs in a single reaction has been practiced for reducing the expense of genotyping a panel of SNPs useful for identity control.

**Findings:**

To further decrease *SNaPshot*'s cost, we introduced the Perl script *SNPmplexViewer*, which facilitates the analysis of trace files for reactions performed without the use of fluorescent size standards. *SNPmplexViewer *automatically aligns reference and target trace electropherograms, run with and without fluorescent size standards, respectively. *SNPmplexViewer *produces a modified target trace file containing a normalised trace in which the reference size standards are embedded. *SNPmplexViewer *also outputs aligned images of the two electropherograms together with a difference profile.

**Conclusions:**

Modified trace files generated by *SNPmplexViewer *enable genotyping of *SnaPshot *reactions performed without fluorescent size standards, using common fragment-sizing software packages. *SNPmplexViewer*'s normalised output may also improve the genotyping software's performance. Thus, *SNPmplexViewer *is a general free tool enabling the reduction of *SNaPshot*'s cost as well as the fast viewing and comparing of trace electropherograms for fragment analysis. *SNPmplexViewer *is available at http://cowry.agri.huji.ac.il/cgi-bin/SNPmplexViewer.cgi.

## Background

Fragment analysis refers to any analysis of genetic markers which relies on variations in the length of a specific DNA sequence to indicate the presence or absence of certain marker alleles. Fragment analyses include, among others, the detection and genotyping of variations in short tandem repeats (STRs) and single nucleotide polymorphisms (SNPs) using fluorescently labelled DNA fragments [1].

DNA-based traceability provides the most reliable way to link a source carcass to meat products by matching genetic marker profiles [2]. Efficient DNA-based traceability schemes require cost-effective genotyping of a limited set of genetic markers that are capable of distinguishing an individual from the rest of the herd. Microsatellite assays for cattle identification are widely used because of the high degree of polymorphism in STR *loci*. Twelve STR *loci *are recommended by the International Society for Animal Genetics (ISAG) for routine use in bovine parentage testing and identification [3]. Six additional microsatellites, which are among the list of *loci *recommended by the Food and Agriculture Organization of the United Nations (FAO) for genetic studies of domestic animals [4], are optionally included in an 18 STR multiplex assay, which is based on fragment analysis [5]. SNPs are used as an alternative to microsatellites in identity control. Another fragment-analysis assay, *SNaPshot*, which is a primer-extension-based method, has been used to multiplex 25 SNPs for traceability assay in cattle [2]. This 25-plex assay detected the extended products using four different fluorochromes and extension primers with oligonucleotide tails of differing lengths. Thus, the concise length of the entire electropherogram was controlled to 81 bases [2].

*SnaPshot *fragment analysis estimates fragment size relative to fluorescently labelled DNA fragments of known length. The DNA fragments are electrophoresed under denaturing conditions in a matrix, which enables size separation at single-nucleotide resolution. The size standards combined with the samples of interest co-migrate in the electrophoretic system, undergo the same electrophoretic forces and provide the necessary data to generate a calibration curve. Based on this curve, the size of unknown fragments in the experimental sample is determined by fragment analysis software [1].

Roughly one-half of the cost of automated genotyping is due to the fluorescently labelled internal size standards, which have relatively few vendors [6]. Hence, minimizing the use of this expensive factor can improve the cost-effectiveness of such fragment analyses. Herein we describe a software solution for electronically embedding the size standards in the trace results obtained from different ABI sequencers (model series 310, 3100 and 3700). Based on the *SeqDoC *algorithm [7], alignment of a reference trace with a target trace, which is run without size standard, places the internal size standards of the reference trace in their expected positions in the target; and allows re-creating the data of the channel for the standards in the target trace. We demonstrate the efficiency of this method in the genotyping of the afore-mentioned 25-plex *SNaPshot *assay for cattle traceability [2].

## Implementation

*SNPmplexViewer *is based on the algorithm implemented in *SeqDoC *[7]. It follows four steps that are similar to those described for *SeqDoC *and an additional step that creates a modified target trace file containing a normalised trace in which the reference size standards are embedded. Each of the steps was modified to handle the FSA format used for fragment analysis, which is more complex than the ABI format handled by *SeqDoC*. As the ABI format is a subtype of the FSA format, *SNPmplexViewer *is compatible with both formats.

### Reading channel data from trace files

Two trace files (FSA or ABI formats), one a reference and the other the target trace, are the main user input. These may be indicated from the command line or uploaded via the web form in the on-line version. The channel data and other relevant data are extracted using the Perl ABIF.pm module [8]. Trace data for the reference size standard is assumed to be recorded in the fifth channel, which is reserved for an emission spectrum matching that of the LIZ™ Size Standard.

### Normalisation and trimming of traces

Since raw data are differently represented by the different sequencer models, data are first transformed according to the sequencer model by either removing control numbers or applying a matrix that accounts for the overlaps in the dye spectrum. To correctly align trace runs with strong and weaker signals, it is necessary to normalise the trace values of the four data channels of the dye-labelled fragments to be sized and remove blank data at the beginning of the trace. To achieve this, *SNPmplexViewer *takes an approach that differs from that of *SeqDoC*, which does consider the average values of signal and noise for each of the channels. To obtain these values, our algorithm first calculates the average signal of all points. Then it estimates the noise to be the mean value of all points that are below the signal average of all points. Subtracting this value from the signal average of all points gives the average data signal.

Normalisation is performed for each channel individually, and sets all of the data points that are below the estimated noise value to zero. The other data points are considered signal peaks and the program scales each of these data points according to the optimal value indicated by the user. In the on-line version, values of 50, 100, 200, 400 or 800 are available. The average data signal is divided by the optimal value to give a scaling factor, and the point being normalised is then scaled by dividing it by this factor.

If use of the 25-plex system option is indicated by the user, the program scans the channels for peaks that may correspond to this system and trims the beginning of the trace accordingly. Otherwise, it uses the first data peak of the first channel as the data starting point and uses the *SeqDoC *algorithm to remove blank trace at the end of each electropherogram by deleting the terminal values from the traces where all channel values are less than 50. In both options, the reference trace guides the search for the starting point in the target trace by limiting the region that is being scanned for peaks to 200 data points ahead of the analogous starting position determined for the reference trace. This solution improves removal of the blank trace at the beginning of the target trace as target traces may often display noise peaks ahead of the real data and these peaks may interfere with proper assignment of the data starting point.

### Alignment of traces

The alignment procedure follows that of the *SeqDoC *algorithm except for one minor modification: the sequences are sampled every three data points and difference scores are calculated for the subsequent 15 data points. If the difference score is reduced by the insertion or deletion of a single data point, then the target traces are adjusted accordingly, by either duplication or removal of the data point at the test position.

### Trace generation, difference estimation and imaging

**Figure 1:**
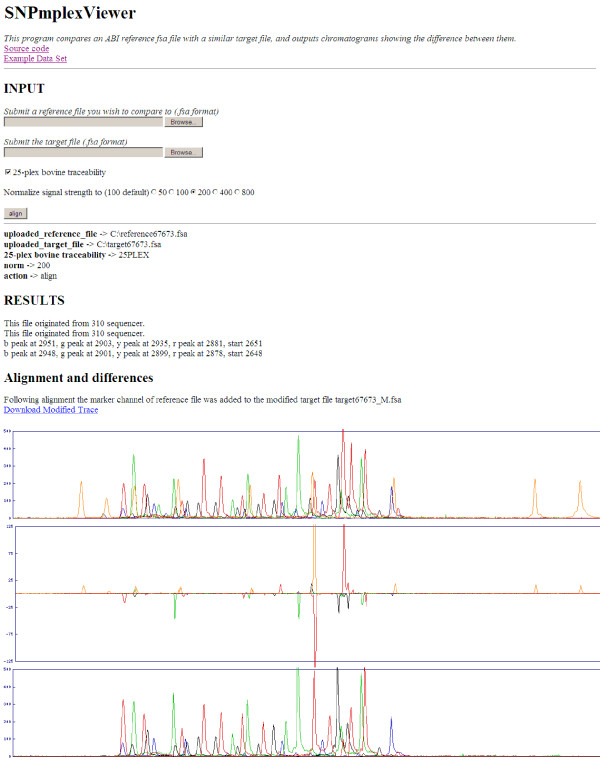
***SNPmplexViewer *web form**. A brief description of the program is followed by web links to the source code and to an example data set and its associated results. The input section contains the two input fields for uploading the reference and target traces and the checked box indicates that the defaults for the 25-plex traceability system should be used. The "200" option is selected in the radio buttons to signify that this was the optimal signal value for the normalisation procedure. Clicking the "align" button submits the form to the server which returns the records of the basic input parameters. The results section first indicates the sequencer identity and the position of the first signal peaks for each of the dyes and the actual scan at which the analysis started after trimming of the blank trace. The link "Download Modified Trace" allows downloading the normalised and modified target trace file in which the reference size standards are embedded. The upper and lower electropherograms are of the reference and target traces, respectively. These were well aligned as indicated by the difference trace between them.

To help the user verify the alignment, the output includes three aligned images: the reference, target and difference-profile electropherograms (Figure 1). The algorithm is essentially identical to that described for *SeqDoC *for the four genotyping channels. Briefly: following normalisation and alignment of the sequences, a 'difference profile' is calculated. For each data point, values of the target trace are subtracted from the equivalent values in the reference trace and the changes are highlighted by squaring the difference value and multiplying the result by the square root of the sum of the differences of other channels which change in the opposite direction. The three images are generated by the Perl GD::Graph module, and are returned to the user in web page format. The difference trace displays bidirectional peaks at the points of base changes between the reference and target traces. *SNPmplexViewer *also adds an orange graph for the fifth channel to the electropherogram of the reference trace, indicating the size standards.

### Creating a normalised target trace file with embedded size standards

Based on the original target file, a modified trace file is generated. Using the Bio::Trace::ABIF module, the data of the four channels of the target trace are replaced with the normalised and aligned data-point values. The raw data from the fifth channel of the reference trace is used to overwrite the fifth channel of the modified target trace file. This operation is ignored if no fifth dye is present in the input traces. For the ABI310 sequencer model, the trace matrix is overwritten by a matrix that indicates that the channels now represent pure dyes.

## Results and Discussion

### The algorithm implemented in SNPmplexViewer

**Figure 2:**
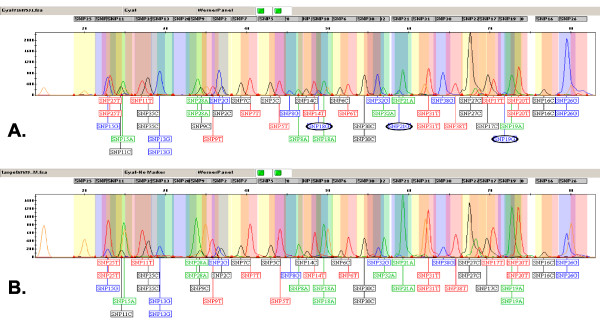
**Fragment analysis of a 25-plex *SNaPshot *reaction without size standards**. *SNaPshot *reaction products of the 25-plex cattle traceability system [2] were separated by capillary electrophoresis on an ABI310 sequencer and analysed with *GeneMapper *software. The genotypes of each SNP and the SNP bins are indicated in boxes below and above the trace, respectively. Genotypes are coloured in green, black, blue and red for A, C, G and T nucleotides, respectively. Bins are shaded following the same colour scheme except for the C bins, which are yellow. (A) Electropherogram from conventional analysis of an individual using GeneScan™ -120 LIZ™ size standard (orange peaks). (B) Electropherogram from the analysis of the same individual with no size standards using a modified trace file, which was obtained as *SNPmplexViewer *output.

Trace electropherograms of fragment analyses such as *SNaPshot *(Figure 2) can be easily interpreted by an experienced human operator, even without viewing the size-standard data. The human mind compares the pattern of the obtained peaks with the expected pattern as observed in previous experiments, and identifies the genotypes according to peak colour, sequence and relative intensity. The algorithm implemented in *SNPmplexViewer *follows a similar concept but instead of genotyping, it embeds indications for the expected location of the size-standard peaks within the trace, which allows further analysis by the genotyping software.

The reference trace provides *SNPmplexViewer *with the equivalent of human memory for the pattern of all expected peaks, including those of the size standard. Thus, this trace file should be obtained from a run of an individual that is heterozygous for all of the SNP markers, or several individuals whose DNA mixture would reveal all possible peaks. The process in which the human mind compares the pattern of the peaks with the expected blueprint is equivalent to the normalisation and alignment steps in *SNPmplexViewer*. The basic difference between a *SNaPshot *trace electropherogram and a sequence trace is that the former is very short and the peaks are mostly ambiguous. Hence, correct trimming of the noise from data channels is critical for the alignment procedure. Unlike the *SeqDoC *algorithm that first trims the blank sequence, *SNPmplexViewer *first estimates the noise level in each channel. We defined noise as the data points with signals that are lower than the mean value of the data points that are below the average signal of all data points. To alleviate the effect of noise on alignment and trimming, all of the noise points are set to zero before the trimming and alignment steps. However, we found that this procedure may not be efficient for traces in which voltage peaks precede the real data (not shown). To better handle such traces, at least in the 25-plex traceability system [2], the program scans the signal peaks for the pattern expected from the first two SNP alleles and trims the trace accordingly.

The alignment step follows the *SeqDoC *algorithm. Data points are added or removed from the target trace in order to align the target peaks with their corresponding reference peaks. Unlike sequence traces, peaks in *SnaPshot *traces are dense and routinely overlap, hence, minor modifications to the *SeqDoC *algorithm were required to achieve a refined alignment: the traces were sampled every three data points, not five, and difference scores were calculated for the subsequent 15 data points and not 30. Following the alignment step, the target peaks are located in positions that agree with the positions of the size-standard peaks in the fifth channel of the reference trace. The transfer of this fifth channel data into the fifth channel of the target file creates a modified target file that is very similar to what would have been obtained if the target fragments had been electrophoresed together with the size standards (Figure 2). It should be noted that traces of microsatellites are not suitable as input for *SNPmplexViewer*. The fragments in these traces display a complex stutter and their sizes cannot be precisely predicted. Therefore, the algorithm is not likely to efficiently align the reference trace with the target trace for this type of traces.

#### Typical output

The output of *SNPmplexViewer *is a modified trace file that includes the electronically embedded size-standards. For allele calling, this file can be further analysed by fragment-sizing software. To demonstrate the analysis of typical output generated by *SNPmplexViewer*, we chose *GeneMapper *[9], which is a leading commercial software package. *GeneMapper *electropherograms comparing a conventional 25-plex trace (Figure 2A) with the output trace from *SNPmplexViewer *(Figure 2B) are shown. Peaks have virtually identical positions in these two electropherograms. The artificially implanted orange size standard peaks are more pronounced in the modified trace (Figure 2B) as a result of the normalisation process and the application of extra volume of this expensive reagent during the preparation of the reference trace. The normalisation process also enhanced the green A-nucleotide peaks but lowered the blue G-nucleotide peaks. This resulted in different callings for the alleles of SNPs 18, 19 and 21 (circled alleles, Figure 2). The calling of the modified trace differed due to the lowering of the G-allele peaks below the detection default limit. All of these changes corresponded to the real status of these SNPs, as validated by other genotyping methods, including direct sequencing and Sequenom MALDI-TOF mass spectrometry. Thus, it is likely that the superfluous G-nucleotide peaks arose from non-specific amplification. It is possible to raise the thresholds for peak detection so that the G-peaks in the conventional trace will be also correctly called. However, such a modification is problematic since other low peaks of other electropherograms that are clearly distinguishable to the human eye would be missed. Hence, besides allowing better alignment of the reference and target traces, the normalisation process implemented in *SNPmplexViewer *enhances the genotyping robustness of GeneMapper.

#### Uploading via the web form

*SNPmplexViewer *can be used locally as a command-line tool by running the Perl script SNPmplexViewer.pl (Additional file 1). The *SNPmplexViewer *web form allows using *SNPmplexViewer *via the internet at http://cowry.agri.huji.ac.il/cgi-bin/SNPmplexViewer.cgi. The upper part of the web form displays a brief description of the program and web links to the source code and to an example data set and its associated results (Figure 1). The user's main input is two ABI trace files (ABI or FSA formats), one a reference and the other the target trace, which can be uploaded using the input fields. Below these fields the user can indicate if the defaults of the 25-plex traceability system should be used. Checking this box will trim the sequence by searching for the pattern of peaks expected from a 25-plex trace, and indicates that the user wishes to produce a modified target trace file in which the reference size standards are embedded. Using the radio buttons, output signal intensities can be controlled by indicating the optimal signal value for the normalisation procedure. Clicking the "align" button submits the form to the server (Figure 1, input section). Employing a simple CGI script (SNPmplexViewer.cgi), the server directs the input to the Perl script (SNPmplexViewer.pl) which, for the record, returns the basic input parameters to the form. In the results section of the form (Figure 1), *SNPmplexViewer *first indicates the sequencer identity and the position of the first signal peaks for each of the dyes followed by the starting point that was set for the analysis in scan units. Data points obtained from scans preceding this scan are trimmed. The link "Download Modified Trace" allows downloading the normalised and modified target trace file in which the reference size standards are embedded. To allow the user to validate the alignment, the *SNPmplexViewer *form generates three electropherograms: the upper and lower electropherograms are of the reference and target traces, respectively. Between these, a difference trace indicates, with large bidirectional peaks, the points of base changes between these traces, following the *SeqDoC *algorithm [7]. However, this algorithm is less efficient for the visualization of genotype differences in the described 25-plex system, as typical differences stem from the presence or absence of bases and not from mutation-driven replacement of one base type with another. Nevertheless, this function makes the *SNPmplexViewer *form handy and the only freely available tool that allows fast viewing of trace files in FSA format, without the need for software installation. To view one file, it is possible to indicate the same file in both reference and target fields. As the FSA format is an extension of the ABI format, it is also possible to compare sequences as described for *SeqDoC *[7]. However, *SeqDoC *is better suited to handling long sequencing traces. Other free FSA viewers *STRand *[10], *Peak Scanner™ *[11] and *FSA2PS *[12] have the disadvantage of requiring prior installation. *FSA2PS *only converts the raw data files of fragment analysis to PostScript images, while *STRand *and *Peak Scanner™ *are fragment-sizing softwares that can manage the allele calling for multiple samples. Hence, *SNPmplexViewer *is the only viewer that allows fast viewing of a single FSA trace or the comparing two traces without setting up a complex project.

## Conclusions

*SNPmplexViewer *is based on the algorithm implemented in *SeqDoC*. It follows the steps of reading channel data from trace files, normalising and trimming the traces, aligning the traces and generating trace and difference images, which help the user validate the alignment. In an additional step, it creates a modified target trace file containing a normalised trace in which the reference size standards are embedded. Two trace files, one a reference and the other the target trace, are the main user input. By transferring the size standard information from the reference trace into the aligned target trace, a modified target trace file is formed. Using further genotyping software, this modified trace allows fragment analysis of traces in the absence of size standards and reducing this analysis cost as well as improved allele calling due to the trace-normalisation process. *SNPmplexViewer *may also be used as a handy free tool for viewing and comparing ABI trace files in the popular FSA format.

## Availability and requirements

Program name: *SNPmplexViewer*

Project home page: http://cowry.agri.huji.ac.il/cgi-bin/SNPmplexViewer.cgi

Source code: http://cowry.agri.huji.ac.il/DATA_SET_SV/SNPmplexViewer_pl.html

Operating system(s): Platform-independent

Programming language: Perl CGI

Other requirements: Requires Perl GD::Graph and Bio::Trace::ABIF modules

License: None for web access, GNU GPL for source code

Any restrictions to use by non-academics: No restrictions

## Additional material

Example data set is available via the project home page.

## List of abbreviations

CGI, common gateway interface; SNP, single nucleotide polymorphism

## Competing interests

The authors declare that they have no competing interests.

## Authors' contributions

YS was responsible for the Perl programming. AS performed the ABI sequencer analyses. ES drafted the manuscript and designed *SNPmplexViewer*. All authors have read and approved the final manuscript.

## Supplementary Material

Additional file 1 ***SNPmplexViewer*****source code**. Perl script in text format. Click here for file

## References

[B1] Podini D Vallone PM SNP genotyping using multiplex single base primer extension assays Methods Mol Biol 2009 578 379 391 10.1007/978-1-60327-411-1_23 19768606

[B2] Karniol B Shirak A Baruch E Singrun C Tal A Cahana A Kam M Skalski Y Brem G Weller JI Ron M Seroussi E Development of a 25-plex SNP assay for traceability in cattle Animal Genetics 2009 40 353 356 10.1111/j.1365-2052.2008.01846.x 19292709

[B3] ISAG 2008 Workshop Report http://www.isag.us/Docs/ISAG2008_CattleParentage.pdf

[B4] Measurement of domestic animal diversity - a review of recent diversity studies ftp://ftp.fao.org/docrep/fao/010/a1250e/annexes/Thematic%20Studies/CGRFA_WG_AnGR_3_04_Inf3.pdf

[B5] van Haeringen G Bovine Genotypes™ Panel 3.1 Instruction manual 2010

[B6] Symonds VV Lloyd AM A simple and inexpensive method for producing fluorescently labelled size standard Molecular Ecology Notes 2004 4 768 771 10.1111/j.1471-8286.2004.00763.x

[B7] Crowe ML SeqDoC: rapid SNP and mutation detection by direct comparison of DNA sequence chromatograms Bmc Bioinformatics 2005 6 133 10.1186/1471-2105-6-133 15927052 PMC1156871

[B8] ABIF http://cpansearch.perl.org/src/VITA/Bio-Trace-ABIF-1.05/lib/Bio/Trace/ABIF.pm

[B9] Product Bulletin GeneMapper® Software v4.1 http://www3.appliedbiosystems.com/cms/groups/mcb_marketing/documents/generaldocuments/cms_077415.pdf

[B10] Toonen RJ Hughes S Increased throughput for fragment analysis on an ABI PRISM (R) automated sequencer using a membrane comb and STRand software Biotechniques 2001 31 1320 1324 11768661

[B11] Peak Scanner™ Software https://products.appliedbiosystems.com/ab/en/US/adirect/ab?cmd=catNavigate2&catID=603624&tab=DetailInfo

[B12] Krawczyk J Goesmann A Nolte R Werber M Weisshaar B Trace2PS and FSA2PS: two software toolkits for converting trace and fsa files to PostScript format Source Code Biol Med 2009 4 4 10.1186/1751-0473-4-4 19622158 PMC2722627

